# Mechanistic physiology-based pharmacokinetic modeling to elucidate vincristine-induced peripheral neuropathy following treatment with novel kinase inhibitors

**DOI:** 10.1007/s00280-021-04302-5

**Published:** 2021-06-02

**Authors:** Venkatesh Pilla Reddy, Adrian J. Fretland, Diansong Zhou, Shringi Sharma, Buyun Chen, Karthick Vishwanathan, Dermot F. McGinnity, Yan Xu, Joseph A. Ware

**Affiliations:** 1 Early Oncology, Oncology Research & Development, AstraZeneca, Cambridge, UK; 2 Clinical Pharmacology and Quantitative Pharmacology, Clinical Pharmacology and Safety Sciences, Biopharmaceuticals Research & Development, AstraZeneca, Cambridge, UK; 3 Early Oncology, Oncology Research & Development, AstraZeneca, Boston, MA USA; 4 Clinical Pharmacology and Quantitative Pharmacology, Clinical Pharmacology and Safety Sciences, Biopharmaceuticals Research & Development, AstraZeneca, Boston, MA USA; 5 Quantitative Clinical Pharmacology, AstraZeneca , South San Francisco, CA USA

**Keywords:** Acalabrutinib, Chemotherapy, Drug interaction, Drug modeling, p glycoprotein, Pharmacokinetics

## Abstract

**Purpose:**

Limited information is available regarding the drug–drug interaction (DDI) potential of molecular targeted agents and rituximab plus cyclophosphamide, doxorubicin (hydroxydaunorubicin), vincristine (Oncovin), and prednisone (R-CHOP) therapy. The addition of the Bruton tyrosine kinase (BTK) inhibitor ibrutinib to R-CHOP therapy results in increased toxicity versus R-CHOP alone, including higher incidence of peripheral neuropathy. Vincristine is a substrate of P-glycoprotein (P-gp, ABCB1); drugs that inhibit P-gp could potentially cause increased toxicity when co-administered with vincristine through DDI. While the combination of the BTK inhibitor acalabrutinib and R-CHOP is being explored clinically, the DDI potential between these therapies is unknown.

**Methods:**

A human mechanistic physiology-based pharmacokinetic (PBPK) model of vincristine following intravenous dosing was developed to predict potential DDI interactions with combination therapy. In vitro absorption, distribution, metabolism, and excretion and in vivo clinical PK parameters informed PBPK model development, which was verified by comparing simulated vincristine concentrations with observed clinical data.

**Results:**

While simulations suggested no DDI between vincristine and ibrutinib or acalabrutinib in plasma, simulated vincristine exposure in muscle tissue was increased in the presence of ibrutinib but not acalabrutinib. Extrapolation of the vincristine mechanistic PBPK model to other P-gp substrates further suggested DDI risk when ibrutinib (area under the concentration–time curve [AUC] ratio: 1.8), but not acalabrutinib (AUC ratio: 0.92), was given orally with venetoclax or digoxin.

**Conclusion:**

Overall, these data suggest low DDI risk between acalabrutinib and P-gp substrates with negligible increase in the potential risk of vincristine-induced peripheral neuropathy when acalabrutinib is added to R-CHOP therapy.

**Supplementary Information:**

The online version contains supplementary material available at 10.1007/s00280-021-04302-5.

## Introduction

Development of novel combination therapies to improve standard-of-care therapy in cancer is complex. Combination therapy with rituximab plus cyclophosphamide, doxorubicin (hydroxydaunorubicin), vincristine (Oncovin), and prednisone (R-CHOP) is often used to treat lymphomas and other types of cancers [1, 2]. R-CHOP is used as front-line treatment in patients with diffuse large-B cell lymphoma (DLBCL), but approximately 40% of these patients have treatment-refractory disease or experience relapse following R-CHOP therapy [3]. While the addition of an approved, molecularly targeted agent has the potential to increase response rates or deepen responses in some patients [3–5], data are limited on the potential for drug–drug interactions (DDIs) with these novel combinations. Such DDIs could result in the development of adverse events (AEs) that may require a reduction in R-CHOP dosage intensity or treatment discontinuation, and may result in poorer outcomes.

**Fig. 1:**
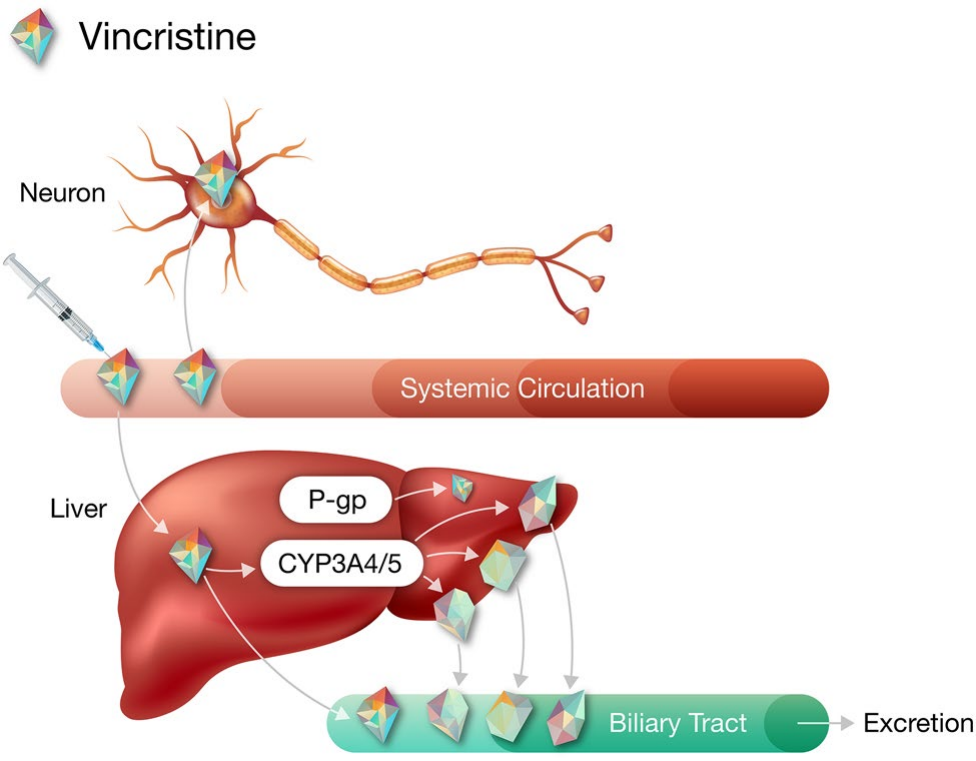
Vincristine pharmacokinetics and pharmacodynamics. Vincristine belongs to the vinca alkaloid class of chemotherapeutics and works by arresting dividing cells in metaphase through binding to the β-subunit of tubulin heterodimers, thereby preventing polymerization into microtubules and causing cellular apoptosis. Following intravenous administration, vincristine passively diffuses throughout the body. In the liver, vincristine is metabolized predominantly by CYP3A5 and excreted; vincristine is also a known substrate of multidrug-resistance (MDR) transporters, including permeability glycoprotein (P-gp). In neurons, microtubules are critical components of nerve fiber axons; vincristine binding to the β-subunit of tubulin can cause a slow, progressive axonal sensorimotor neuropathy, called vincristine-induced peripheral neuropathy (VIPN). Adapted with permission from Mora et al. [17]

Several studies are investigating the combination of oral Bruton tyrosine kinase (BTK) inhibitors with R-CHOP [5–14]. Acalabrutinib is a next-generation, potent, highly selective, covalent small-molecule inhibitor of BTK approved in adults with previously treated mantle cell lymphoma and in patients with chronic lymphocytic leukemia or small lymphocytic lymphoma [15]. The addition of acalabrutinib to R-CHOP is currently being investigated in patients with DLBCL (NCT03571308, NCT04002947) [11, 14]. In a phase 3 trial of the BTK inhibitor ibrutinib added to R-CHOP therapy in treatment-naïve patients with non-germinal center B cell-like or activated B cell-like DLBCL, greater toxicity was observed with ibrutinib plus R-CHOP compared with placebo plus R-CHOP (incidence of serious AEs: 53.1% vs 34.0%), leading to a higher incidence of treatment discontinuation due to AEs (31.5% vs 13.6%) [5]. Rates of discontinuation of any component of R-CHOP were also higher in the ibrutinib plus R-CHOP arm (26.7% vs 11.7% with placebo plus R-CHOP) due to higher incidences of several AEs including peripheral neuropathy [5]. Vincristine-induced peripheral neuropathy (VIPN) is a known, potentially dose-limiting side effect of intravenous (IV) vincristine that can severely affect patient quality of life [16]. While the exact mechanism of the observed increase in VIPN with combination treatment has not been determined, vincristine is a known substrate of multidrug-resistance transporters, including p-glycoprotein (P-gp, ABCB1), and is metabolized predominantly by enzymes in the CYP3A subfamily (Fig. 1) [17, 18]. When administered with the CYP3A4 and P-gp inhibitor nifedipine, vincristine plasma area under the curve (AUC) is increased 3.4-fold [19, 20]. Ibrutinib has been demonstrated to inhibit P-gp transport at clinical doses [21]; therefore, ibrutinib-induced inhibition of P-gp in motor neurons could potentially increase exposure to vincristine, though DDI between ibrutinib and vincristine remains to be explored in muscle. While acalabrutinib and its major active metabolite ACP-5862 have not been shown to inhibit P-gp at clinically relevant concentrations [15], it is important to determine if there is an increased risk of VIPN with the addition of acalabrutinib to R-CHOP therapy. Clinical trials are needed to directly assess the incidence of VIPN with acalabrutinib plus R-CHOP regimens and, as previously mentioned, two such studies are currently ongoing (NCT03571308, NCT04002947) [11, 14]; however, model simulation can be used to predict DDIs between acalabrutinib and vincristine [22, 23].

The aim of this study was to build a human physiology-based pharmacokinetic (PBPK) model of vincristine using in vitro absorption, distribution, metabolism, and excretion (ADME) and in vivo clinical PK parameters. The PBPK model was verified by matching with observed clinical vincristine plasma PK profiles following intravenous administration and by comparing the likely predicted impact of vincristine administration on extensive and poor CYP3A5 metabolizers versus observed concentrations. Finally, the verified model was used prospectively to predict the outcomes of interactions with molecularly targeted agents, including ibrutinib and acalabrutinib, in plasma and muscle tissue to predict potential DDI with combination therapy that could result in increased toxicity.

## Methods

### In vitro cell-based and vesicular transport assays

Cell-based and inside-out vesicle assays were conducted to determine the inhibition constant (IC_50_) for acalabrutinib and its active metabolite ACP-5862 using vincristine or N-methyl-quinidine as P-gp probes. The IC_50_ for ibrutinib inhibition of P-gp has been previously established using cell-based assays [24].

Cell-based assays assessed the bidirectional transport of vincristine by P-gp across Caco-2 cells in the absence and presence of acalabrutinib or ACP-5862. These cells were cultured in supplemented Eagle’s minimal essential medium in a humidified culture chamber (37 ± 2 °C, 95 ± 5% relative humidity, and 5 ± 1% CO_2_) on a porous membrane in 24-well transwell plates for 21 days before the experiment until they formed a confluent monolayer with tight junctions. After cell culture, trans-epithelial electrical resistance values were measured and cells were preincubated at 37 ± 2 °C for 30–60 min. Following preincubation, digoxin (10 µM)-containing medium and solvent control, control inhibitor (valspodar 1 µM), or acalabrutinib (3, 10, 30, 100, 300, 400 µM) or ACP-5862 (0.3, 1, 3, 10, 30, 50 µM) was added to the donor (apical) chamber and medium-containing solvent control, control inhibitor (valspodar 1 µM), or acalabrutinib (3, 10, 30, 100, 300, 400 µM) or ACP-5862 (0.3, 1, 3, 10, 30, 50 µM) was added to the receiver (basolateral) chamber. Samples were collected from the receiver chamber after 120 min. In wells in which recovery was calculated, samples were taken from the donor chamber at the start of the incubation and after 120 min. Samples containing vincristine were mixed with internal standards and analyzed by liquid chromatography tandem mass spectrometry (see Supplementary Materials). When more than 50% inhibition was observed, an additional experiment was performed to determine the IC_50_ value. IC_50_ values were determined from the decrease in efflux ratios or uptake rates (percentage of control) in the presence of inhibitors and was calculated according to the following equation, where slope equals Hill factor (unitless):

$$Y \, = {\text{ minimum }} + \, \left( {{\text{maximum }}{-}{\text{ minimum}}} \right)/\left( {1 \, + \, \left[ {{\text{concentration}}/{\text{IC}}_{50} } \right]^{{{\text{slope}}}} } \right)$$

Vesicular transport assays assessing P-gp-dependent uptake of ^3^H-vincristine were performed using inside-out membrane vesicles prepared from HEK293 cells overexpressing human P-gp, with control cells that do not overexpress P-gp serving as negative controls. Assays were conducted in 96-well plates with a total protein content of 50 µg/well per membrane vesicle preparation. All assays were conducted in the presence of adenosine triphosphate (ATP) or adenylyl-imidodiphosphate (AMP-PNP), a nonhydrolyzable analog to distinguish between ATP-dependent transporter-mediated uptake and passive diffusion into vesicles. Incubation with ^3^H-*N*-methyl-quinidine (^3^H-NMQ) 1 µM in a solvent control provided a positive control for P-gp activity. The transport of ^3^H-vincristine 1 µM was first tested at eight incubation time points (0.5–20 min) to determine the optimal incubation time; eight concentrations (0.13–200 μM) of ^3^H-vincristine were then tested at the optimal incubation time of 2 min to determine the kinetic parameters (K_m_ and V_max_) of P-gp-mediated vincristine transport. All reaction mixtures were preincubated for 15 min at 37 ± 1 °C (or 32 ± 1 °C for ^3^H-NMQ), initiated by the addition of pre-warmed 12 mM MgATP (Mg is a cofactor for ATP) or 12 mM AMP in assay buffer as a background control, and stopped by the addition of ice-cold washing buffer and immediately mounted to 96-well filter plates. The vincristine concentration in each sample was determined by liquid scintillation counting after the plate was dried.

Vesicular transport inhibition assays were then conducted in the presence of BTK inhibitors to assess inhibition of P-gp-mediated vincristine transport. Acalabrutinib (0.04–30 µM with vincristine and 0.14–100 µM with ^3^H-NMQ), ACP-5862 (0.01–6.25 µM), and ibrutinib (0.04–30 µM) were incubated with membrane vesicle preparations (total protein: 50 µg/well) and the tritium-labeled probe substrate (^3^H-NMQ 1 µM or ^3^H-vincristine). The concentration ranges tested were selected based on the solubility limit in the assay buffer(s). Incubations were conducted in the presence of 4 mM ATP or AMP to distinguish between transporter-mediated uptake and passive diffusion into the vesicles. The P-gp inhibitor valsopodar 1 µM served as a positive control for inhibition. Reactions were stopped by the addition of ice-cold washing buffer and immediately mounted to 96-well filter plates. The vincristine concentration in each sample was determined by liquid scintillation counting after the plate was dried. The amount of translocated probe substrate was determined in counts per minute. ATP-dependent transport (pmol/mg protein/min) was calculated for each set of assay conditions by subtracting the calculated accumulation values in AMP-containing assays (background activity values) from the calculated accumulation values in ATP-containing assays (see Supplementary Materials). IC_50_ values were derived from a four-parametric logistic equation (log[inhibitor] versus response minus variable slope); the curve was fitted to the relative activity versus vincristine concentration plot using non-linear regression based on the following equation where *X* equals the log of concentration; *Y* equals the response, decreasing as *X* increases; “top” equals the maximal response in the same units as *Y*; “bottom” equals the maximally inhibited response in the same units as *Y*; logIC_50_ uses the same log units as *X*; and “Hillslope” is the Hill slope factor Hill slope (unitless):

$$Y \, = {\text{ bottom }} + \, \left( {{\text{top }}{-}{\text{ bottom}}} \right)/\left( {1 \, + \, 10^{{[\left( {{\text{LogIC}}50 - X} \right)*{\text{Hill slope}}]}} } \right)$$

The maximal response and maximally inhibited response values were not constrained to constant values of 100 and 0, respectively, unless otherwise noted.

### Mechanistic PBPK model development

Physicochemical parameters, in vitro parameters for absorption and metabolism, and concentration time data from clinical studies [5, 25, 26] were used to develop the vincristine mechanistic PBPK model in Simcyp version 19 (Simcyp Limited, Sheffield, UK), accounting for an interplay between metabolism by CYP3A subfamily enzymes, polymorphic CYP3A5, P-gp transport, and disease. The specific input parameters used for the vincristine PBPK model generation are listed in Table 1 [5, 26]. A three-stage system including drug-specific parameters, system-specific parameters, and trial design characteristics was utilized by the Simcyp software for PBPK model development, as previously described [22, 23]. The renal clearance value used in the input parameters for the vincristine PBPK model of 4.18 L/h (observed value) was estimated as ~ 12% of the total clearance listed in the product information, based on the published PK information for vincristine [27]. For modeling, the P-gp transport kinetics in the liver and kidney were used to estimate the renal clearance. The estimated renal clearance based on the PBPK model was 3.3 L/h, which is within 1.3-fold of the observed value. Because Madin-Darby canine kidney (MDCK) cell lines were used to determine the efflux ratios, the Simcyp default relative activity factor/relative expression factor (RAF/REF) for MDCK cells of 1.5 was used for the input parameter. Whole organ clearance and enzyme kinetics for poor and extensive CYP3A5 metabolizers were obtained from Lee and colleagues [28]. A fivefold difference between values in patients with high and low CYP3A expression was assumed. A sensitivity analysis using a ~ 30-fold lower P-gp inhibition IC_50_ value (0.16 µM instead of 5 µM) was also performed for ibrutinib and acalabrutinib, as per European Medicines Agency (EMA) PBPK guidance to investigate the uncertainty in the measured IC_50_ values of P-gp inhibition [29].

**Table 1:** Input parameters for vincristine physiology-based pharmacokinetic model

Parameter	Value	Source
Molecular weight, g/mol	824.96	Product information
Log P	2.67	Internal data
Compound type	Monoprotic acid	Internal data
pKa	5.15 (acid)	O’Neil MJ (ed). The Merck Index
B/P	1.2	Internal data
Fu, plasma	0.51	Internal data
Distribution model	Full PBPK	
V_SS_, L/kg	1.64	Based on Sethi et al. [26]^a^
Elimination model		
fu_mic_	0.75	Predicted within Simcyp simulator
V_max_ (extensive CYP3A5 expressors), pmol/min/mg protein	416	DIDB
V_max_ (poor CYP3A5 expressors), pmol/min/mg protein	114	DIDB
K_m_ (high CYP3A5 expressors), µM	18.5	DIDB
K_m_ (low CYP3A5 expressors), µM	89.8	DIDB
V_max_ (CYP3A4), pmol/min/mg protein	0.90	DIDB
K_m_ (CYP3A4), µM	19.5	DIDB
Permeability limited liver model		
CL_PD_, µl/min/million hepatocytes/kidney/muscle	0.37/0.1/0.1	Estimated based on the physiochemical properties of vincristine
fu_IW_ liver/muscle	0.036/0.0771	Predicted within Simcyp simulator
fu_EW_ liver/muscle	1/0.80	Predicted within Simcyp simulator
Transporter kinetics in liver, kidney, muscle	P-gp	
*J*_max_, pmol/min/million cells	77	DIDB
K_m_, µM	17.1	DIDB
RAF/REF	1.5	Set to 1.5^b^
Trial design and simulation settings based on Younes et al. [5]		
Population	Sim-Cancer	Virtual population
Number of trials	5	
Subject/trial	5	
Age range (years)	20–90 years	
Proportion of females	0.47	

Additional system parameters used for the permeability-limited model developed for muscle are shown in Supplemental Fig. 3

CL_PD_: passive diffusion clearance; DIDB: University of Washington Drug Interaction Database, https://www.druginteractionsolutions.org/, accessed June 2019; Fu: unbound fraction; fu_EW_: unbound fraction (extracellular water); fu_IW_: unbound fraction (intracellular water); fu_mic_: unbound fraction in microsomal system; J_max_: rate of transport; K_m_: rate of metabolism; MDCK: Madin-Darby canine kidney; P-gp: permeability glycoprotein; RAF/REF: relative activity factor/relative expression factor; V_max_: maximum rate of metabolism; *V*_ss_: volume of distribution

^a^Method 2 (Roger and Rowland method) was used to estimate *V*_ss_ and matched to the observed clinical value derived from Sethi et al. via KP scalar

^b^Because MDCK cell lines were used to determine kinetic parameters, the RAF/REF was set to the Simcyp default for MDCK cells of 1.5

### Simulation of vincristine plasma concentration–time profiles

Vincristine plasma concentration–time profiles were simulated using the PBPK model developed with in vitro metabolism data (Table 1) to account for CYP3A5 status and P-gp-mediated efflux, which were compared with observed vincristine concentrations of cancer patients (*N* = 25; five patients/five trials [NCT01855750, NCT01236391, NCT01599949, NCT01646021, NCT01569750]) with poor or extensive CYP3A5 metabolism. A virtual oncology population based on the population described by Schwenger et al. [30] was used for the simulations. Demographics for the virtual population were matched with inclusion criteria of the five DDI trials, and simulations were performed as per clinical trial designs. A default frequency of 0.83 within the PBPK simulator was used for patients with the CYP3A5 non-expressor genotype (*3/*3; CYP3A5 poor metabolizers), using a 2-mg dose of vincristine infused over 15 min based on the published PK study design of Younes et al. [5] or Villikka et al. [25]. Vincristine PBPK model simulations were also performed to assess the effects of coadministration of itraconazole to verify P-gp contributions to vincristine excretion. Itraconazole is an azole anti-fungal drug and a known inhibitor of both CYP3A4/5 and P-gp [31]. The input parameters for vincristine PBPK model simulations including coadministration with itraconazole and hydroxy-itraconazole are provided in Supplemental Table 1.

### Vincristine drug–drug interaction simulations in plasma and muscle

The ability of the developed mechanistic PBPK model to predict the DDI of vincristine with BTK inhibitors was tested at the level of systemic circulation and in muscle tissue. Vincristine plasma concentrations were simulated after a single IV dose of 2 mg infused over 15 min to cancer patients in the presence of ibrutinib 560 mg QD at steady state. Similarly, vincristine muscle concentrations were simulated after a single IV dose of 2 mg to cancer patients in the presence or absence of multiple doses of ibrutinib or acalabrutinib. The current version of Simcyp software does not have a permeability-limited model for simulations in muscle. Therefore, a generic organ program using a permeability model option within Simcyp version 19 software was used to generate hypotheses including increased vincristine exposure by calibrating the parameters for efflux transporters to mimic the inhibiting effect. No changes were made to remove the existing muscle compartment; thus, the permeability-limited model essentially duplicated the muscle compartment. While simulation of tissue concentrations using this fit-for-purpose model may not be fully mechanistic and could potentially reduce the biological relevance of the permeability-limited model, it could be useful for hypothesis generation. All simulations using the vincristine PBPK model were carried out using the input parameters for poor CYP3A5 metabolizers (CYP3A5 genotype *3/*3 status) to mimic a worst-case scenario (i.e., a cumulative effect of no CYP3A5 expression and inhibition of P-gp and CYP3A4 in the patient population).

### Mechanistic PBPK model application

Additional mechanistic PBPK models were used to assess several DDI scenarios with other P-gp substrates. The venetoclax PBPK model was developed based on a hybrid of published and simulated data [32, 33], and the digoxin PBPK models were derived from the Simcyp compound library. The hybrid venetoclax PBPK model was verified as a monotherapy in the fasted and fed states, with known CYP3A modulators rifampicin in healthy volunteers and ketoconazole in patients with chronic lymphocytic leukemia, and with digoxin (Supplemental Fig. 2A–C). In vitro, venetoclax is a substrate of P-gp with net efflux ratio of 13 per Caco-2 transwell assay [34], and the prescribing information for this drug suggests avoiding concomitant use of venetoclax with a strong or moderate P-gp inhibitor [34, 35]. Digoxin is a substrate of both intestinal and renal P-gp and has a weak affinity for P-gp, thus making it a useful probe substrate drug when investigating potential P-gp inhibitors [36]. Published PBPK models of ibrutinib and acalabrutinib were employed to understand in vitro/in vivo extrapolation in predicting DDI when co-dosed with vincristine in cancer patients [37, 38]. Additional verification was performed using available venetoclax and ibrutinib clinical DDI data [39]. The sources and drug-specific input parameters for the PBPK models listed above are provided in Supplemental Table 1.

## Results

### In vitro cell-based and vesicular transport assays

In both cell-based and inside-out vesicle in vitro systems, ibrutinib was observed to strongly inhibit P-gp, with IC_50_ values of 5 µM and 6 µM, respectively. Acalabrutinib demonstrated weak P-gp inhibition, with IC_50_ values of 98 µM in the cell-based system and 57 µM in the vesicle system, and its major active metabolite, ACP-5862, demonstrated no P-gp inhibition at IC_50_ values up to 50 µM in both systems.

### Vincristine mechanistic PBPK model verification

**Fig. 2:**
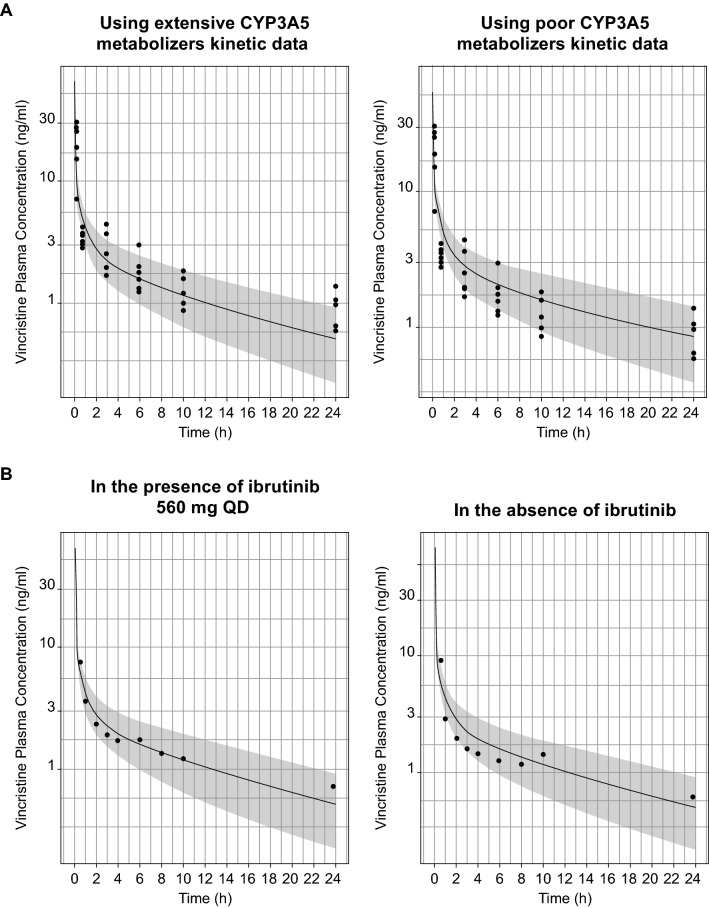
Vincristine plasma concentrations after a single oral dose of 2 mg IV infusion to cancer patients (**A**) with poor or extensive CYP3A5 metabolism and (**B**) in the presence and absence of ibrutinib treatment. The solid lines represent the mean vincristine plasma concentration predicted by the mechanistic PBPK model. The shaded areas show the 90% prediction interval of the simulations. The black dots represent the observed individual plasma concentrations of cancer patients of unknown genotype. BID: twice daily; IV: intravenous; PBPK: physiology-based pharmacokinetic; QD: once daily

Data from the basic static equation used to inform DDI risk for vincristine are included in Supplementary Materials. Based on simulations using the vincristine PBPK model, the predicted percentage fraction metabolized (%fm) was 32%, the predicted percentage fraction excreted (%fe) was 68%, the median half-life of vincristine in poor CYP3A5 metabolizers was 79 h (range 64–96), and the median half-life in extensive CYP3A5 metabolizers was 18 h (range 11–25). Simulated vincristine plasma concentration–time profiles in extensive and poor CYP3A5 metabolizers appeared to be reasonably well predicted by the developed PBPK model compared with observed vincristine concentrations in a population of cancer patients with unknown CYP3A5 genotype (Fig. 2A). Additionally, when the simulated vincristine concentration–time profiles were compared with observed clinical data in cancer patients following vincristine dosing [25], reasonable agreement (within 1.25-fold) was observed for AUC and observed clearance (Table 2 [5, 25]). This finding supports the translatability of the vincristine mechanistic PBPK model for use in simulating the DDI between P-gp inhibitors and vincristine as a victim drug. The results of simulations assessing the contributions of P-gp versus CYP3A4/5 inhibition by itraconazole to vincristine excretion demonstrated contributions due to both P-gp and CYP3A4/5 inhibition (Supplemental Table 2). Based on our simulations, we hypothesize that P-gp contributes approximately 37% to the 68% of vincristine that is eliminated, with the remaining vincristine elimination mediated by unknown route, and CYP3A-mediated metabolism accounting for 32% of the overall elimination.

**Table 2:** Comparison of simulated vincristine PK with observed plasma PK at a dose of 2 mg after IV dosing in cancer patients

	Vincristine monotherapy		
	Simulated Mean ± SD	Observed Mean ± SD^a^	Observed/simulated ratio
AUC_0-∞_ (ng h/ml)	52.2 ± 17.8	65.1 ± 10.1	1.25
V_d_ (L/kg)	8.05 ± 2.2	12.8 ± 2.2	1.60
CL (ml/min)	666 ± 200	569 ± 76	0.85

	Vincristine DDI with ibrutinib at a 560-mg dose		
	Simulated Mean ± SD	Observed Mean ± SD^b^	Observed/simulated ratio
AUC_0-24 h_ ratio	1.18 ± 0.06	0.89	0.75
C_max_ ratio	1.08 ± 0.03	1.26	1.16

AUC: area under the curve; CL: clearance; *C*_max_: peak serum concentration; NCA: non-compartmental analysis; *V*_d_: volume of distribution

^a^As reported in Villikka et al. [25]

^b^Calculated NCA parameters based on Younes et al. [5]

### Simulating drug–drug interactions

In the DDI simulation of vincristine administered in the presence of ibrutinib, vincristine plasma concentration–time profiles derived from the mechanistic PBPK model generally recovered the range of vincristine PK profiles and PK parameters within two-fold of the observed values in the presence of ibrutinib, with a simulated AUC ratio of 1.18 consistent with the observed AUC ratio of 0.89 (Fig. 2B and Table 2) and demonstrated no clinically meaningful interaction at the systemic plasma level due to P-gp inhibition by ibrutinib.

**Fig. 3:**
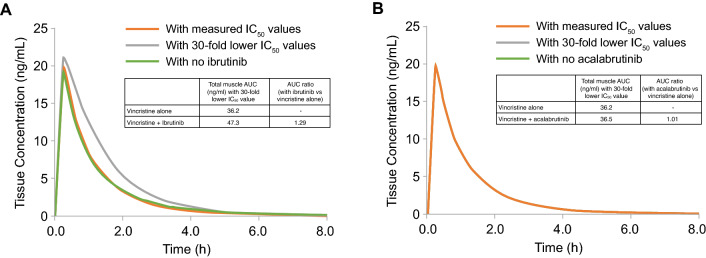
Vincristine muscle concentrations simulated using measured IC_50_ values and IC_50_ values 30 times lower than observed values in the presence or absence of **A** ibrutinib or **B** acalabrutinib. In panel B, the orange, gray, and green lines are overlapping. The orange line indicates concentrations in the presence of BTKi using measured IC_50_ values. The gray line indicates concentrations in the presence of BTKi using IC_50_ values 30 times lower than the observed values. The green line indicates concentrations in the absence of BTKi. AUC, area under the concentration–time curve; BTKi, Bruton tyrosine kinase inhibitor

When observed IC_50_ values were used to simulate vincristine muscle tissue concentrations in the presence and absence of ibrutinib and acalabrutinib, no substantial changes in vincristine exposure were observed (Fig. 3). When ibrutinib and acalabrutinib IC_50_ values 30 times lower than the observed IC_50_ values (included as a sensitivity analysis) were used to generate the vincristine simulations, substantially increased concentrations were noted in the presence of ibrutinib up to 4 h after dosing compared with the simulation generated in the absence of ibrutinib, while minimal differences in vincristine concentration were observed in the presence and absence of acalabrutinib. The simulated vincristine muscle tissue exposure increased by > 30% (AUC with interaction: 47 ng/ml*h; AUC without interaction: 36 ng/ml*h) when the P-gp IC_50_ value of ibrutinib used to generate the simulation was 30 times lower than the lowest of the original input values (Fig. 3A). Simulations using a 30-fold lower IC_50_ value of acalabrutinib in the model input parameters showed no to minimal changes in the simulated vincristine muscle concentration (AUC with and without interaction: 36 ng/ml*h; Fig. 3B).

### PBPK modeling with other P-gp substrates

**Fig. 4:**
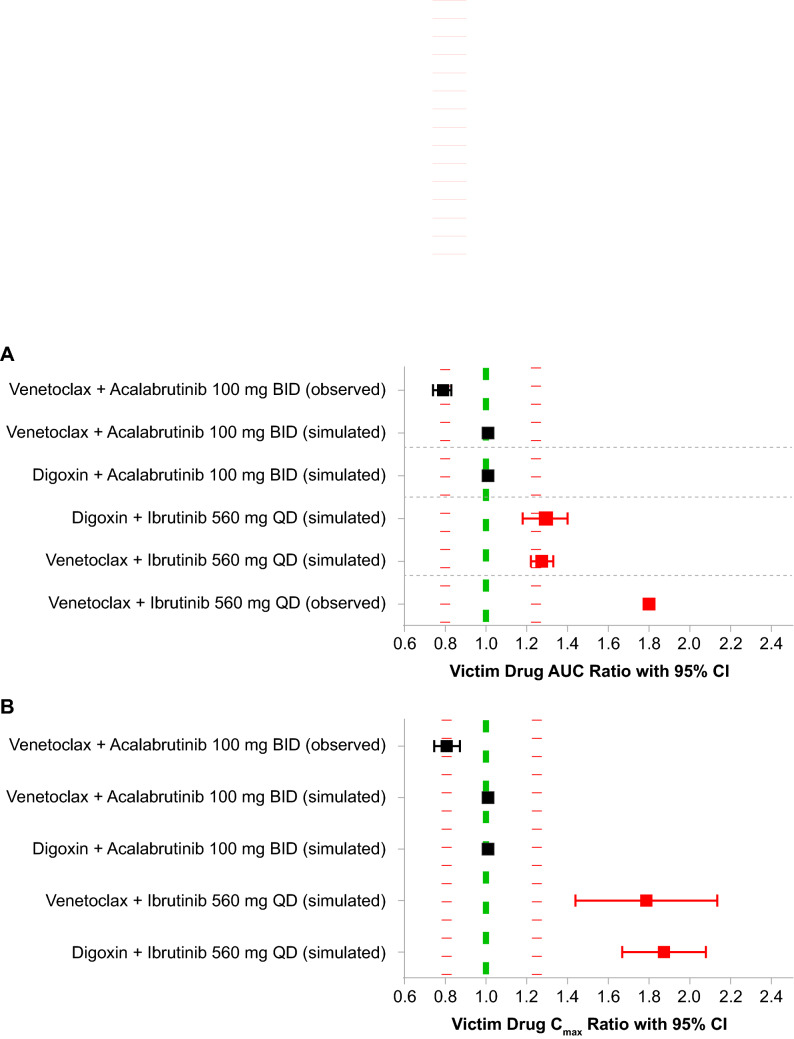
Summary of geometric means of **A** AUC and **B** *C*_max_ ratios in DDI simulations for other P-gp substrates with ibrutinib and acalabrutinib in plasma. Area between the two dashed red lines indicates no DDI effect. AUC: area under the curve; BID: twice daily; CI: confidence interval; C_max_: maximum concentration; DDI: drug–drug interaction; P-gp: permeability glycoprotein; QD: once daily

When venetoclax administration was simulated in the presence of ibrutinib 560 mg QD, plasma exposure to venetoclax increased approximately 1.2- to 1.8-fold (Fig. 4). Similar results were observed when digoxin administration was simulated in the presence of ibrutinib, with digoxin exposure also increasing approximately 1.2- to 1.8-fold. However, when venetoclax or digoxin administration was simulated in the presence of acalabrutinib 100 mg BID, no increase in substrate plasma exposure was noted. These simulations were substantiated by observed data demonstrating a 1.8-fold increase in venetoclax plasma AUC ratio in the presence of ibrutinib [39] and a slight decrease in venetoclax AUC and C_max_ ratios in the presence of acalabrutinib, fairly comparable to the results observed in the simulations (Fig. 4). Additionally, simulations using a 30-fold lower P-gp IC_50_ value for venetoclax in the model input parameters showed a 1.2-fold increase in total venetoclax muscle tissue concentrations at *C*_max_ in the presence of ibrutinib while no changes in venetoclax muscle tissue exposure were noted in the presence of acalabrutinib (Supplemental Fig. 1).

## Discussion

The utility of mechanistic PBPK modeling in predicting DDIs is well established, and the predictability of models containing P-gp components are continuing to improve as new in vitro and in vivo data become available [23]. In the current study, a mechanistic PBPK model for vincristine was developed using observed in vitro ADME and in vivo clinical PK parameters and was verified by comparing the likely predicted impact of vincristine administration on extensive and poor CYP3A5 metabolizers versus observed concentrations. The verified PBPK model was able to predict a vincristine PK profile in the presence of ibrutinib and suggested no DDI at the systemic plasma level between vincristine and ibrutinib. However, simulations in muscle tissue using an IC_50_ value 30 times lower than the observed value suggested increased vincristine muscle tissue exposure in the presence of ibrutinib but not acalabrutinib, suggesting potentially different DDI risk levels between the BTK inhibitors at the muscle level. Finally, the simulations generated for the vincristine mechanistic PBPK model were verified with P-gp inhibitors using observed IC_50_ values in plasma (venetoclax and digoxin) and 30-fold lower IC_50_ values in muscle (venetoclax) and suggested a potential DDI risk with ibrutinib, but not acalabrutinib. Overall, these data suggest that the risk of DDI between acalabrutinib and P-gp substrates is low and indeed lower than for ibrutinib and P-gp substrates; therefore, based on the hypotheses and simulations explored in this analysis, the risk of VIPN is likely not increased with the addition of acalabrutinib to R-CHOP therapy.

In the in vitro transport assays used to inform the vincristine mechanistic PBPK model, ibrutinib, but not acalabrutinib or ACP-5862, inhibited P-gp. Of note, while the in vitro assays utilized two different systems (inside-out membrane vesicles prepared from mammalian cells stably transfected to overexpress P-gp, or Caco-2 cells), the results from both in vitro systems were generally consistent. Basic static equation analyses developed to predict P-gp-mediated interactions using observed IC_50_ values further flagged a potential DDI risk with clinically relevant doses of ibrutinib due to P-gp inhibition at the gut level, while no DDI risk was flagged with clinically relevant doses of acalabrutinib. These findings are supported by the prescribing information for the two BTK inhibitors, which report P-gp transport inhibition with ibrutinib but not with acalabrutinib [15, 21].

The developed vincristine mechanistic PBPK model also suggests that vincristine is a victim drug of P-gp inhibitors, as vincristine concentrations are predicted to increase with concomitant use of drugs that inhibit P-gp in muscle tissue. Moreover, simulations indicated that vincristine concentrations decreased more rapidly over time in extensive CYP3A5 metabolizers, in line with the observed clinical data following vincristine dosing [40, 41]. The vincristine PBPK model could not be verified due to the lack of clinical data necessary to definitively corroborate the simulations. However, these results support the translatability of the developed vincristine model results to physiologically relevant scenarios and provide further confidence in its ability to predict DDI risks based on the hypotheses explored in this analysis.

As expected, simulations of vincristine plasma concentrations in the presence and absence of clinically relevant doses of ibrutinib suggested no DDI at the systemic plasma level due to P-gp inhibition. These simulations support previous reports suggesting no interaction between vincristine and ibrutinib at the plasma level [5]. It should be noted that while the PBPK model simulated the effect of P-gp inhibitors on systemic venetoclax exposure, it does not fully capture the extent of the inhibition nor fully capture the clinical observations. We hypothesize that the differences in P-gp inhibition at the plasma level (no increase in exposure to vincristine when administered with ibrutinib but an increase in venetoclax and digoxin exposure when administered with ibrutinib) are due to the differences in their routes of administration. Because P-gp expression is relatively enriched in the gastrointestinal tract [42], IV administration of vincristine may circumvent P-gp inhibition observed with orally administered drugs such as venetoclax and digoxin. Intestinal P-gp induction is known to decrease systemic exposure and may attenuate both the absorption fraction and absorption rate [43]; therefore, intestinal P-gp inhibition via orally administered drugs may be expected to increase systemic exposure. The developed PBPK model reasonably predicted the range of vincristine PK profiles and PK parameters compared with observed values. However, vincristine muscle concentrations were substantially higher for several hours following ibrutinib but not acalabrutinib dosing when IC_50_ values 30 times lower than the observed values were used, suggesting a potential DDI risk for ibrutinib but not acalabrutinib at the muscle tissue level. Although the absolute risk of ibrutinib-mediated P-gp DDI was only recapitulated in the PBPK model when a P-gp IC_50_ value lower than the experimentally determined value was used, these simulations can inform the relative risk of acalabrutinib DDI via this mechanism, which appears to be substantially lower than the DDI risk with ibrutinib based on these simulations. The differences in P-gp inhibition at the plasma and muscle levels could potentially be due to the relatively low expression of P-gp in skeletal tissue [44, 45]; with fewer transporters available, lower concentrations of inhibitor may have a larger impact on total protein efflux rate or capacity. Because increasing vincristine AUC levels have been shown to correlate with the degree of neurotoxicity [46], the increases in AUC in muscle tissue demonstrated in the current study may be clinically relevant.

Our simulations indicate that vincristine muscle concentrations increase following ibrutinib administration when a low P-gp IC_50_ is used, which is consistent with the observation of an increased incidence of peripheral neuropathy, potentially due to vincristine, in patients receiving ibrutinib and R-CHOP combination therapy [5]. While the exact mechanism of VIPN is unknown, vincristine induces cellular apoptosis by binding to microtubules and preventing completion of mitosis in dividing cells [17]. Vincristine is a substrate of the efflux transporter P-gp [17]; thus, ibrutinib-induced inhibition of P-gp would presumably increase vincristine cellular concentrations, leading to increased apoptosis. No clinically meaningful DDI was demonstrated at the systemic plasma level due to P-gp inhibition by ibrutinib, but our simulations indicate that vincristine muscle concentrations increase following ibrutinib administration. Therefore, we hypothesize that the ibrutinib-mediated increase in VIPN is the result of tissue-level effects and not mediated through systemic-level interactions.

Additionally, our simulations indicated that vincristine concentrations decreased more slowly over time in poor CYP3A5 metabolizers. This simulation is also consistent with the fact that vincristine is metabolized by enzymes in the CYP3A subfamily [18]. Indeed, low CYP3A5 expression has been associated with an increased risk of VIPN in children with acute lymphoblastic leukemia [40]. These combined data and simulations suggest the potential for an increased risk of DDI-induced VIPN with ibrutinib or other P-gp inhibitors and R-CHOP combination treatment in patients homozygous for the CYP3A5 non-expressor genotype (*3/*3). Moreover, CYP3A5 expression is highly variable among different ethnic populations, with the CYP3A5*3 variant allele present in approximately 85 to 98% of European Americans and in approximately 27–48% of African Americans [47]. Care should be taken to determine appropriate combination dosing regimens in patients with low CYP3A5 expression to limit the potential for VIPN.

Additional PBPK models further suggest an increased risk of DDI with ibrutinib compared with acalabrutinib when dosed with other P-gp substrates. The simulated DDI between ibrutinib and venetoclax in the current study confirms the PK results of a prior study of ibrutinib plus venetoclax in patients with chronic lymphocytic leukemia or small lymphocytic leukemia (NCT02910583) in which venetoclax AUC was higher when coadministered with ibrutinib compared with historical data for single-agent venetoclax [39]. However, caution should be used when interpreting the observed data showing DDI with different agent combinations in Fig. 4, as the data are derived from clinical studies, not dedicated DDI studies. As such, several aspects of the source studies that could potentially affect drug metabolism may not be well captured or may not be available. To our knowledge, clinical data on AE incidence with venetoclax administered in combination with ibrutinib compared with venetoclax alone are not available. Similar results were generated when interactions between the P-gp substrate digoxin and ibrutinib were simulated. However, when venetoclax or digoxin administration was simulated in the presence of acalabrutinib 100 mg BID, no increase in substrate exposure was observed, suggesting minimal DDI risk when acalabrutinib is given concomitantly with P-gp substrates. These simulations also confirm the PK results of an ongoing study of acalabrutinib in combination with venetoclax and obinutuzumab or rituximab in patients with treatment-naïve or relapsed/refractory chronic lymphocytic leukemia (NCT02296918) in which no increase in the venetoclax peak serum concentration or AUC ratio was observed in combination with acalabrutinib, as shown in Fig. 4.

Based on these overall results, there is no reason to anticipate an increased risk of VIPN in patients treated with acalabrutinib and R-CHOP. Further studies examining the effects of vincristine binding to tubulin [48] and on vincristine distribution and DDI will be explored to more fully elucidate factors increasing the risk of VIPN. Additionally, the two ongoing studies assessing acalabrutinib in combination with R-CHOP regimens (NCT03571308, NCT04002947) will provide further information regarding the safety profile of therapies combining acalabrutinib and vincristine.

## Conclusions

Prospective simulations using a vincristine mechanistic PBPK model are potentially useful for identifying drug combinations leading to a greater risk of VIPN due to P-gp inhibition. Our modeling suggests that vincristine muscle tissue concentrations increase when dosed with the P-gp inhibitor ibrutinib but not with acalabrutinib. Additional modeling suggests that the subsequent risk of VIPN with vincristine concentrations could be even higher in patients who are homozygous for the CYP3A5 non-expressor genotype (*3/*3) following combination treatment with R-CHOP and a P-gp inhibitor. Based on these results, no increased risk of VIPN with acalabrutinib and R-CHOP combination therapy is expected. Extrapolation of the developed model to other P-gp substrates suggests a potential DDI risk when venetoclax is coadministered with ibrutinib, but not with acalabrutinib.

## Supplementary Information

Below is the link to the electronic supplementary material.

Supplementary file1 (PDF 404 KB)

Supplementary file1 (xlsx 21 KB)

Supplementary file1 (xlsx 22 KB)

## Data Availability

Data underlying the findings described in this manuscript may be obtained in accordance with AstraZeneca’s data sharing policy described at https://astrazenecagrouptrials.pharmacm.com/ST/Submission/Disclosure.
