# Supplementary material for: Mechanistic physiology-based pharmacokinetic modeling to elucidate vincristine-induced peripheral neuropathy following treatment with novel kinase inhibitors
Source: Cancer Chemother Pharmacol. 2021 Jun 2;88(3):451–64. doi: 10.1007/s00280-021-04302-5 (PMC8316236; doi:10.1007/s00280-021-04302-5)
Supplement: Supplementary file 1 — Supplementary file1 (PDF 404 KB) [file 280_2021_4302_MOESM1_ESM.pdf]

Supplementary Materials to:

Mechanistic Physiology-Based Pharmacokinetic Modeling to Elucidate Vincristine-Induced Peripheral Neuropathy Following Treatment With Novel Kinase Inhibitors

Cancer Chemotherapy and Pharmacology

Venkatesh Pilla Reddy,[1] Adrian J. Fretland,[2] Diansong Zhou,[3] Shringi Sharma,[3] Buyun Chen,[3] Karthick Vishwanathan,[3] Dermot McGinnity,[1] Yan Xu,[3] Joseph Ware[3]

[1]Early Oncology, Oncology Research & Development, AstraZeneca, UK; [2]Early Oncology, Oncology Research & Development, AstraZeneca, USA; [3]Clinical Pharmacology and Quantitative Pharmacology, Clinical Pharmacology and Safety Sciences, Biopharmaceuticals Research & Development, AstraZeneca, USA

Corresponding author: Dr. Venkatesh Pilla Reddy

Modelling and Simulation, Early Oncology, Oncology R&D Hodgkin Building,

Chesterford Science Park, Little Chesterford, Cambridge, CB10 1XL, UK

Email: Venkatesh.Reddy@astrazeneca.com

Methods

Bidirectional permeability analyses

All bidirectional permeability analyses were performed with liquid chromatography tandem mass spectrometry methods using an AB SCIEX mass spectrometer with Shimadzu liquid chromatography pumps and autosampler systems. Authentic standards were used, and deuterated analytes were used as internal standards. Assay components are shown in **Supplemental Table 3**.

Probe substrates were quantified using the simplest appropriate weighting and regression algorithm. The regression fit was based on the peak area ratio of the analyte to the internal standard calculated from the calibration standard samples. Stock standard solutions and working solutions were prepared according to the custom Tecan script EVO Std-QC Spiking Solution Prep. Chromatographic peaks were integrated with Analyst Instrument Control and Data Processing Software (AB SCIEX, Ontario, Canada; version 1.6.1).

ATP-dependent accumulation

In vesicular transport substrate assays, the adenosine triphosphate (ATP)-dependent transport of the test article as well as the ATP-dependent fold accumulation were calculated for each concentration and time point in both the transporter-containing and control vesicles using the following equations, where n_{ATP} equals the amount of translocated test article in the presence of 4 mM ATP in pmol/mg and $n_{AMP\text{-}PNP}$ equals the amount of translocated test article in the presence of 4 mM adenylyl-imidodiphosphate (AMP-PNP), in pmol/mg:

$$ATP - dependent\ transport = n_{ATP} - n_{AMP\text{-}PNP}$$

$$Fold\ accumulation = n_{ATP} / n_{AMP\text{-}PNP}$$

If the ATP-dependent fold accumulation value is >2 in transporter containing vesicles, is not observed in the control vesicles, and can be inhibited by a known inhibitor of the transporter, then the test article can be considered a substrate of the transporter investigated.

ATP-dependent transport and relative inhibition (%)

For all wells in the vesicular transport inhibition assays, the amount of the translocated probe substrate was determined in counts per minute (cpm) and ATP-dependent transport (pmol/mg protein/min) was calculated for each concentration using the following formula, where Acc_{ATP} equals probe substrate accumulation with ATP in cpm, Acc_{AMP} equals probe substrate accumulation with AMP-PNP in cpm, TCPM equals cpm in dosing solution, V equals volume per well in µL, CC_{sub} equals probe substrate concentration in µM, Prot equals total protein per well in mg, and t equals incubation time in minutes:

$$ATP - dependent\ accumulation = \left(\left(\frac{Acc_{ATP}}{TCPM}\right) \times V \times CCsub \Big/ \left(\frac{Prot}{1000}\right) / t\right) - \left(\left(\frac{Acc_{AMP}}{TCPM}\right) \times V \times CCsub \Big/ \left(\frac{Prot}{1000}\right) / t\right)$$

Relative ATP-dependent transport (%) values were calculated using the following equation, where A equals the amount of translocated substrate in the presence of test article and ATP, B equals the amount of translocated substrate in the presence of TA and AMP-PNP, C equals the amount of translocated substrate in the presence of

solvent and ATP, and D equals the amount of translocated substrate in the presence of solvent and AMP-PNP:

$$Relative\ ATP\text{-}dependent\ transport\ \% = (A\text{-}B)/(C\text{-}D)*100$$

Relative inhibition (%) was calculated by setting the probe substrate transport value in the absence of test article equal to 100% and was calculated using the following equation, where $Acc_{specific,\ x}$ equals transporter specific accumulation for a given sample in pmol/mg/min and $Acc_{specific,\ vehicle}$ equals transporter specific accumulation for a solvent control in pmol/mg/min:

$$Relative\ inhibition = (Acc_{specific,\ x} / Acc_{specific,\ vehicle})*100\%$$

Basic static equation to determine DDI risk for vincristine

Basic static models were developed to predict the effects of ibrutinib and acalabrutinib on vincristine DDI using the following equations, where R_1 or $R_{1,gut}$ equals the predicted ratios of the victim drug's AUC in the presence and absence of the inhibitor, $I_{max,u}$ equals the maximal unbound plasma concentration of the interacting drug, I_{gut} equals the intestinal luminal concentration of the interacting drug (calculated as the dose/250 ml), and K_i (assumed to be equal to IC_{50}) equals the unbound inhibition constant determined *in vitro* with I and K_i expressed in the same molar concentration unit:

$$R_1 = 1 + (I_{max,u} / K_i)\ and\ R_{1,gut} = 1 + (I_{gut} / K_i)$$

Results

Basic static equation to determine DDI risk for vincristine

Results of the basic static equation analysis suggested P-gp inhibition at the gut level (theoretical maximal gastrointestinal concentration $[I_2]/IC_{50} \geq 10$) but not the hepatic level ($[I_{total}]/IC_{50} \geq 0.1$) with ibrutinib 560 mg QD, while no inhibition was observed with acalabrutinib 100 mg BID at both the gut ($[I_2]/IC_{50} < 10$) and biliary/hepatic (mean unbound steady-state maximum concentration following administration of the highest clinical dose $[I_1]/IC_{50} \leq 0.1$) levels. These results were further complemented with dynamic mechanistic PBPK analyses as detailed in the Methods.

Supplemental Table 1. Input parameters for acalabrutinib, digoxin, venetoclax, ibrutinib, itraconazole, and hydroxy-itraconazole PBPK models

**Parameter**	**Description**	**Units**	**Drugs**					
**Physicochemical (main references)**			**Acalabrutinib** [1]	**Digoxin** (Simcyp Library)	**Venetoclax** (hybrid model based on Emami Riedmaier et al., Freise et al. [2,3])[a]	**Ibrutinib** [4]	**Itraconazole** (Simcyp Library)	**OH-Hydroxy Itraconazole** (Simcyp Library)
MW	Molecular weight	g/mol	465.5	780.94	868.44	440.5	705.6	721.7
log P	Octanol:buffer partition coefficient	–	2.03	1.26	8	3.97	4.47	4.47
pKa	Dissociation constant	–	3.54<br>5.77<br>(diprotic base)	Neutral	3.4<br>10.3<br>(ampholyte)	3.78<br>(monoprotic base)	4.28<br>(monoprotic base)	4.28<br>(monoprotic base)
B:P	Blood-to-plasma partition ratio	–	0.787	1.07	0.6	0.827	0.58	0.58
fu	Fraction unbound in plasma	–	0.026	0.71	0.00085	0.027	0.016	0.016
ka	Absorption rate constant	1/h	1.65	Fa=0.84; ADAM model	Fa=0.73; ADAM model	Fa=1; PPT rate=0.41; ADAM model	Ka=1.5	–
fa	Fraction available from oral dosage form	–	0.98	–	0.73	0.99	1	–
fu_{gut}	Unbound fraction of drug in gut enterocytes (fu_{plasma}/B:P)	–	0.026	1	1	0.11	0.016	–
Pcaco-2 (6.5:7.4)	Caco-2 permeability	x 10^{-6} cm/s (unless otherwise specified)	5.39	12.7	0.28	22.9/19.2 (propranolol)	7.73 x 10^{-4} cm/s (mech P_{eff})[b]	8.01 x 10^{-4} cm/s (mech P_{eff})[b]
**Distribution**								
Vss	Distribution volume at steady state	L/kg	0.21	6.13	0.21	11	2.508	1.03

**Elimination**								
CL/F	Oral clearance	L/h	–	–	–	14.3	84.8	–
CL_{renal}	Renal clearance	L/h	1.33	9.66	–	0.00365	–	–
CL_{int} (based on retrograde) or rCYP assay or HLM or heps	*In vitro* human liver microsomal protein intrinsic clearance	µL/min/ mg protein	rCYP3A4 CL_{int}=9.63; CYP3A4 V_{max}=4.13; Km=2.78; Additional HLM (µL/minutes/ mg)=289.5	Hep as additional CL =0.37	CYP3A4 V_{max}=15; Km=29.4	CYP3A4= 8312; HLM other CL_{int}=364.4	CYP1A2=1; CYP3A4 V_{max}=0.065; Km=0.0039	CYP3A4 V_{max}=0.13; Km=0.027
Transport	Intestine/liver/kidney transporter kinetics	–	–	Jmax=434; Km=177 µM	$CL_{int,T}$ (µL/min/cm^2)= 2.45	–	–	–
**Interaction Parameters**								
Inhibition[c]	Concentration of inhibitor that causes half maximal inhibition (Ki)	µM	CYP3A4/5 (Ki)=23.9 µM; TKI (Ki)=10.1; K_{inact}=1.11 (1/h); P-gp=98 µM; $Fu_{,mic}$=0.97	–	CYP3A4/5 (Ki)=3.6 µM; $Fu_{,mic}$=0.002	CYP3A4/5 (Ki)=7.6 µM; P-gp=6 µM; $Fu_{,mic}$=0.047	CYP3A4/5 (Ki)= 0.0013 µM; P-gp=0.24 µM; $Fu_{,mic}$=1	CYP3A4/5 (Ki)= 0.0023 µM; P-gp=0.24 µM; $Fu_{,mic}$=1

[a]V_{max} and KM values were obtained from the venetoclax PBPK model developed by Freise et al [3]; all other values were obtained from the venetoclax PBPK model developed by Enami Riedmaier et al [2].
[b]Mechanistic P_{eff} model within Simcyp software was used to predict the P_{eff} if no measured Pcaco-2 is available.
[c]P-gp inhibition values were added to the PBPK models based on in vitro internal data.
ADAM, Advanced Dissolution Absorption and Metabolism; Fu_{mic}, unbound fraction in microsomes; Fu_{plasma}, unbound fraction of drug in plasma; HLM, human liver microsomes; J_{max}, efflux rate; K_{inact}, maximal potential rate of inactivation; Km, substrate affinity; P_{eff}, effective permeability; PPT, precipitation rate; rCYP, recombinant cytochrome; TKI, tyrosine kinase inhibitor; V_{max}, maximum initial velocity.

Supplemental Table 2. Simulated effect on vincristine exposure of CYP3A4/5 plus P-gp inhibition versus P-gp inhibition alone

**Vincristine + Itraconazole and Hydroxy-itraconazole**	**AUC Ratio Change (95% CI)**
With CYP3A4/5[a] + P-gp inhibition[b]	2.84 (2.28–3.53)
With only P-gp inhibition[b]	1.80 (1.45–2.24)

[a]Ki=0.0013 μM for itraconazole; Ki=0.0023 μM for hydroxy-itraconazole.
[b]Ki=0.24 μM for both itraconazole and hydroxy-itraconazole.
Ki=concentration of inhibitor that causes half maximal inhibition.

Supplemental Table 3. Components of bidirectional permeability analyses

Transporter	Probe Substrate	Internal Standard	Internal Standard Stock Concentration (Ng/Ml)[a]	Mass Spectrometer	Electrospray Ionization Mode	HPLC Column[b]
P-gp	Digoxin	Digoxin-d_3	1000	4000 QTrap	Positive	Waters Atlantis dC28 (5 µm, 100 x 2.1 mm)

[a]This concentration is diluted 2.67-fold when added to the stopped incubation mixture.
[b]All HPLC columns were preceded by a Phenomenex Luna C-8 guard column (4 × 2.0 mm).
HPLC, high-performance liquid chromatography; P-gp, permeability glycoprotein.

Supplemental Figure 1. Venetoclax muscle concentrations simulated using IC_{50} values 30 times lower than observed values in the presence or absence of (A) ibrutinib or (B) acalabrutinib.

A.

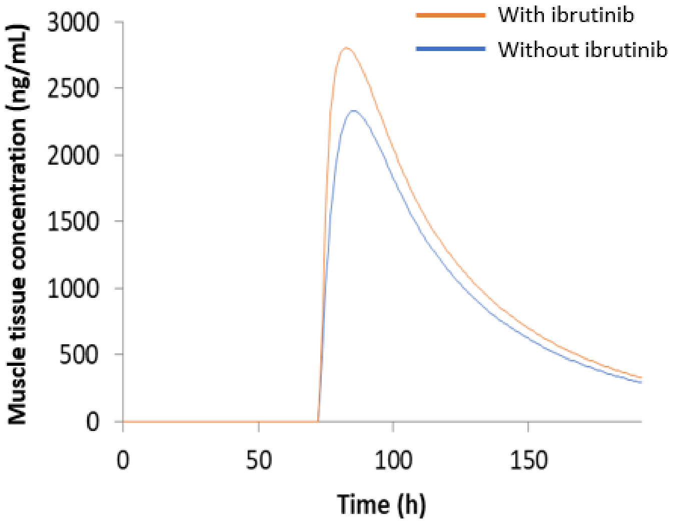

B.

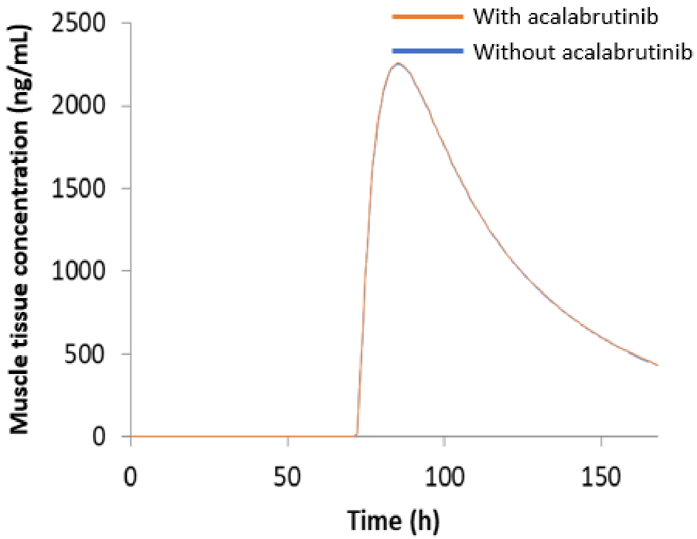

The blue line indicates the simulated C_{max} of venetoclax in muscle tissue, and the orange line indicates the simulated C_{max} of venetoclax in muscle tissue in the presence of BTKi.
C_{max}, maximal concentration; BTKi, Bruton tyrosine kinase inhibitor.

Supplemental Figure 2. Verification of the venetoclax PBPK model (A) in the fasted and fed states following administration of one dose of 100 mg venetoclax to healthy volunteers, (B) with CYP3A modulators rifampicin (in addition to venetoclax 200 mg in healthy volunteers) and ketoconazole (in addition to venetoclax 50 mg QD in patients with non-Hodgkin lymphoma) following a low-fat meal, and (C) with a single oral dose of P-gp inhibitor digoxin 0.5 mg in addition to a single dose of venetoclax 100 mg in healthy volunteers.

A.

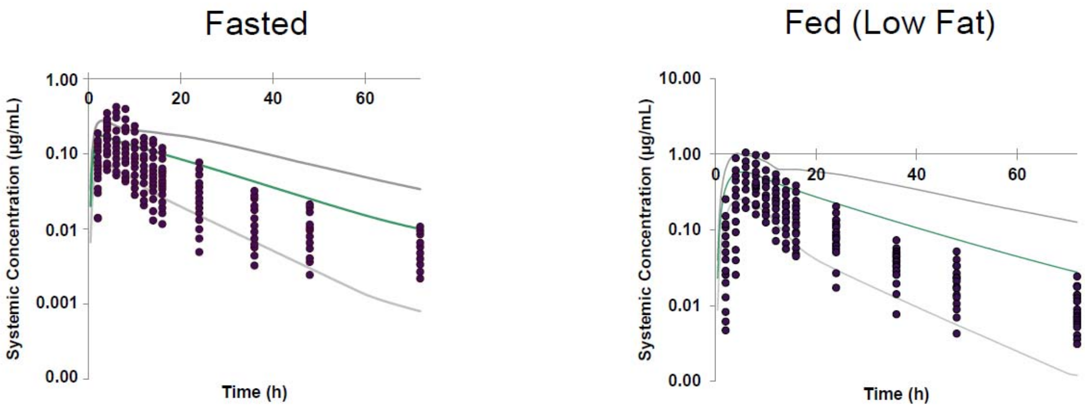

Parameter	Observed (GM)[a]	Predicted (GM)	Ratio (Pred/Obs)
$AUC_{0-\infty}$ (µg/mL/h)	2.25	3.6	1.56
C_{max} (µg/mL)	0.16	0.15	0.93
t_{max} (h)	4.37	3.07	0.70

Parameter	Observed (GM)[a]	Predicted (GM)	Ratio (Pred/Obs)
$AUC_{0-\infty}$ (µg/mL/h)	7.1	10.7	1.50
C_{max} (µg/mL)	0.5	0.58	1.16
t_{max} (h)	6.1	5.4	0.9

B.

Rifampicin 600 mg QD

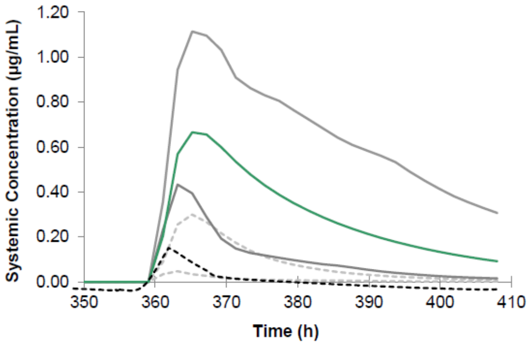

DDI Ratio	Observed (GM)[a]	Predicted (GM±SD)	Ratio (Pred/Obs)
$AUC_{0-\infty}$	0.2	0.13 ± 0.07	0.65
C_{max}	0.3	0.27 ± 0.13	0.9

Ketoconazole 400 mg QD

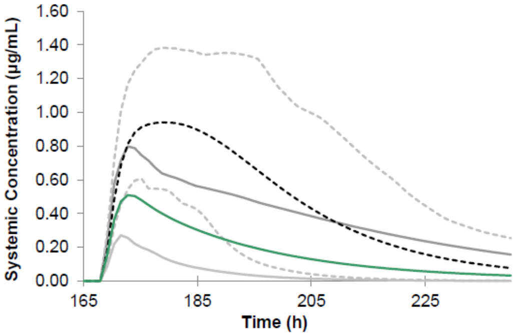

DDI Ratio	Observed (GM)[a]	Predicted (GM±SD)	Ratio (Pred/Obs)
$AUC_{0-\infty}$	6.40	4.18 ± 2.11	0.65
C_{max}	2.32	1.93 ± 0.47	0.83

C.

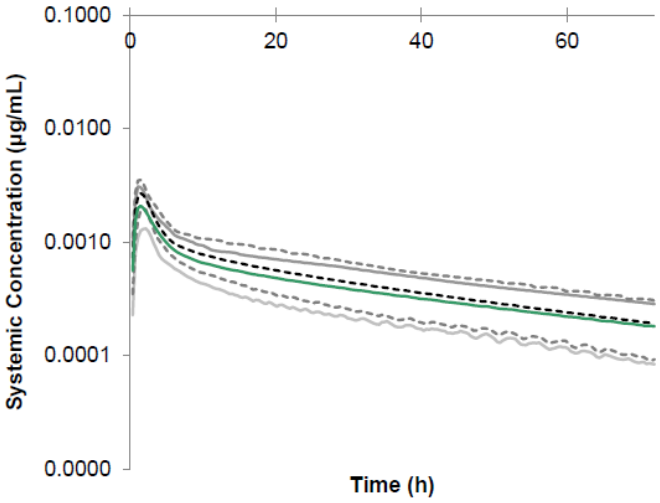

DDI Ratio	Observed (GM [90% CI])[b]	Predicted (GM [90% CI])	Ratio (Pred/Obs)
$AUC_{0-\infty}$	1.09 (0.99–1.19)	1.17 (1.13–1.20)	1.07
C_{max}	1.35 (1.15–1.58)	1.31 (1.26–1.36)	0.96

[a]From Riedmaier et al. [2].
[b]From Chiney et al. [5].
Simulations using ketoconazole took into account the effects of P-gp inhibition (P-gp = 0.42 µM). Simulations using rifampicin did not account for P-gp induction, as the methodology is not yet well established. Purple circles indicate observed values in individual subjects. Solid green lines indicate mean values without interaction, dashed black lines indicate mean values with interaction, solid gray lines indicate 95% prediction intervals without interaction, and dashed gray lines indicate 95% prediction intervals with interaction.

$AUC_{0-\infty}$, area under the concentration-time curve from time zero to infinity; CI, confidence interval; C_{max}, maximal concentration; GM, geometric mean; P-gp, permeability glycoprotein; Pred/Obs, predicted/observed; QD, once daily; SD, standard deviation; t_{max}, time to maximal concentration.

Supplemental Figure 3. Additional system parameters used for the permeability-limited model developed for muscle.

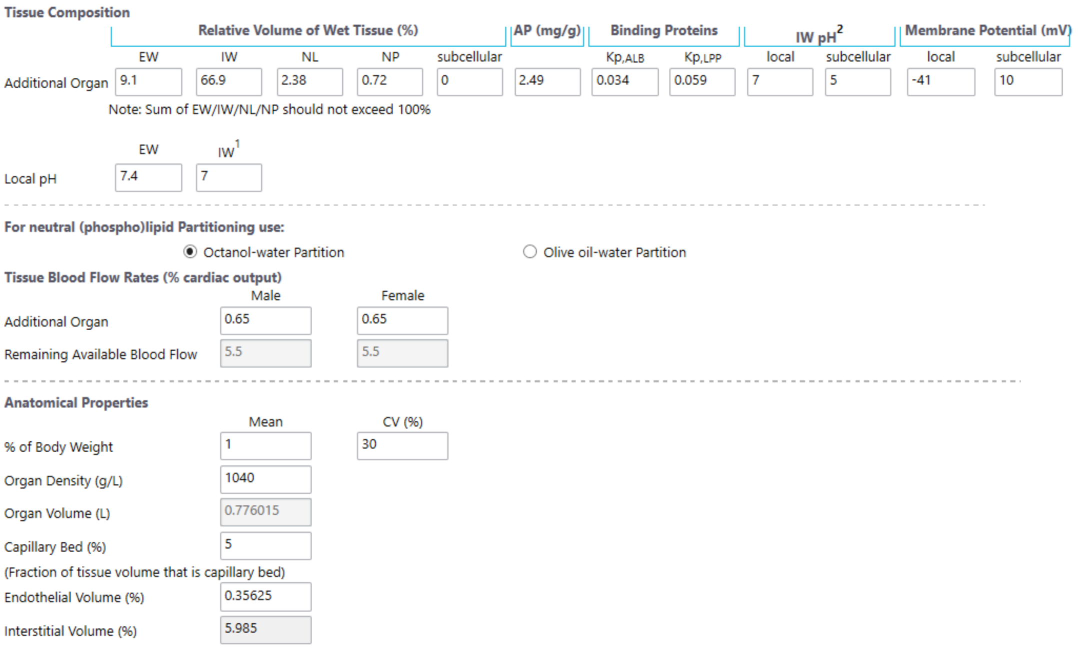

Tissue Composition

	Relative Volume of Wet Tissue (%)					AP (mg/g)	Binding Proteins		IW pH[2]		Membrane Potential (mV)	
	EW	IW	NL	NP	subcellular		Kp,ALB	Kp,LPP	local	subcellular	local	subcellular
Additional Organ	9.1	66.9	2.38	0.72	0	2.49	0.034	0.059	7	5	-41	10

Note: Sum of EW/IW/NL/NP should not exceed 100%

	EW	IW[1]
Local pH	7.4	7

For neutral (phospho)lipid Partitioning use:

(•) Octanol-water Partition ( ) Olive oil-water Partition

Tissue Blood Flow Rates (% cardiac output)

	Male	Female
Additional Organ	0.65	0.65
Remaining Available Blood Flow	5.5	5.5

Anatomical Properties

	Mean	CV (%)
% of Body Weight	1	30
Organ Density (g/L)	1040	
Organ Volume (L)	0.776015	
Capillary Bed (%) (Fraction of tissue volume that is capillary bed)	5	
Endothelial Volume (%)	0.35625	
Interstitial Volume (%)	5.985	

AP, acidic phospholipids; CV, coefficient of variation; EW, extracellular water; IW, intracellular water; Kp_{ALB}, tissue to plasma albumin ratio; Kp_{LPP}, tissue to plasma lipoprotein ratio; NL, neutral lipids; NP, neutral phospholipids.

References

1. Zhou D, Podoll T, Xu Y, Moorthy G, Vishwanathan K, Ware J, Slatter JG, Al-Huniti N (2019) Evaluation of the drug-drug interaction potential of acalabrutinib and its active metabolite, ACP-5862, using a physiologically-based pharmacokinetic modeling approach. CPT Pharmacometrics Syst Pharmacol 8 (7):489-499. doi:10.1002/psp4.12408

2. Emami Riedmaier A, Lindley DJ, Hall JA, Castleberry S, Slade RT, Stuart P, Carr RA, Borchardt TB, Bow DAJ, Nijsen M (2018) Mechanistic physiologically based pharmacokinetic modeling of the dissolution and food effect of a

biopharmaceutics classification system IV compound-the venetoclax story. J Pharm Sci 107 (1):495-502. doi:10.1016/j.xphs.2017.09.027

3. Freise KJ, Shebley M, Salem AH (2017) Quantitative prediction of the effect of CYP3A inhibitors and inducers on venetoclax pharmacokinetics using a physiologically based pharmacokinetic model. J Clin Pharmacol 57 (6):796-804. doi:10.1002/jcph.858
4. de Zwart L, Snoeys J, De Jong J, Sukbuntherng J, Mannaert E, Monshouwer M (2016) Ibrutinib dosing strategies based on interaction potential of CYP3A4 perpetrators using physiologically based pharmacokinetic modeling. Clin Pharmacol Ther 100 (5):548-557. doi:10.1002/cpt.419
5. Chiney MS, Menon RM, Bueno OF, Tong B, Salem AH (2018) Clinical evaluation of P-glycoprotein inhibition by venetoclax: a drug interaction study with digoxin. Xenobiotica 48 (9):904-910. doi:10.1080/00498254.2017.1381779
